# Calcium dysregulation links ALS defective proteins and motor neuron selective vulnerability

**DOI:** 10.3389/fncel.2015.00225

**Published:** 2015-06-16

**Authors:** Sónia S. Leal, Cláudio M. Gomes

**Affiliations:** ^1^Faculdade de Ciências, Biosystems and Integrative Sciences Institute and Department of Chemistry and Biochemistry, Universidade de LisboaCampo Grande, Lisboa, Portugal; ^2^Instituto Tecnologia Química e Biológica, Universidade Nova de LisboaOeiras, Portugal

**Keywords:** neurodegenerative diseases, calcium homeostasis, ALS, proteinopathies, SOD1

## Abstract

More than 20 distinct gene loci have so far been implicated in amyotrophic lateral sclerosis (ALS), a fatal neurodegenerative disorder characterized by progressive neurodegeneration of motor neurons (MN) and death. Most of this distinct set of ALS-related proteins undergoes toxic deposition specifically in MN for reasons which remain unclear. Here we overview a recent body of evidence indicative that mutations in ALS-related proteins can disrupt fundamental Ca^2+^ signalling pathways in MN, and that Ca^2+^ itself impacts both directly or indirectly in many ALS critical proteins and cellular processes that result in MN neurodegeneration. We argue that the inherent vulnerability of MN to dysregulation of intracellular Ca^2+^ is deeply associated with discriminating pathogenicity and aberrant crosstalk of most of the critical proteins involved in ALS. Overall, Ca^2+^ deregulation in MN is at the cornerstone of different ALS processes and is likely one of the factors contributing to the selective susceptibility of these cells to this particular neurodegenerative disease.

## Sporadic and Familial ALS Aggregates Share Identical Proteins

Amyotrophic lateral sclerosis (ALS) is a fatal neurodegenerative disease characterized by the selective degeneration of motor neurons (MN) in the spinal cord, brainstem and cerebral cortex (Rowland and Shneider, 2001). Most cases of ALS are sporadic (sALS) with no known genetic linkage, while approximately 10% are associated with familial forms (fALS), presenting mutations in over 20 genes encoding for distinct proteins with varied functions (Table 1). Despite the heterogeneous genetics of fALS and unknown etiology of sALS and its clear multifactorial character, most ALS patients present similar phenotypes with formation of cytoplasmic proteinaceous aggregates in the affected MN (Al-Chalabi et al., 2012). Notably, many proteins involved in fALS forms are also found in sALS toxic aggregates (Maekawa et al., 2009; Deng et al., 2010; Forsberg et al., 2010; Blokhuis et al., 2013) and found to cross-talk and impact on each other in ALS pathology (Kanekura et al., 2004; Volkening et al., 2009; Tudor et al., 2010; Nihei et al., 2012; Pokrishevsky et al., 2012; Stoica et al., 2014; Osaka et al., 2015). This suggests that apart from mutations, additional chemical and/or biological factors influence the selective involvement of these proteins also in sALS neurodegeneration. In agreement, most of the proteins which are involved in ALS are ordinarily expressed in many distinct cell tissues other than the nervous system (e.g., SOD1, FUS, TDP-43, VAPB, matrin-3, ataxin-2, alsin) but are only found to generate toxicity among MN. The selective vulnerability of such cells suggests that environmental triggers within those neurons are mandatory for the onset of ALS.

**Table 1:** **Heterogeneity of fALS causative genes**.

Protein (% prevalence)	Gene/Protein	Function
FTD/ALS (40–50%) (DeJesus-Hernandez et al., 2011)	*C9ORF72*	Suggested to regulate endosomal trafficking and autophagy
ALS 1 (20%) (Rosen et al., 1993)	*SOD (SOD1)*	Antioxidant; scavenging of superoxide
ALS 10 (5%) (Sreedharan et al., 2008)	*TARDBP (TDP-43)*	RNA and DNA regulation
ALS 6 (4%) (Vance et al., 2009)	*FUS (FUS)*	RNA and DNA regulation
ALS 13 (>1%) (Elden et al., 2010)	*ATXN2 (Ataxin-2)*	Suggested to regulate RNA processing
Other (less prevalent fALS) (Chen et al., 2004; Nishimura et al., 2004; Johnson et al., 2010; Maruyama et al., 2010; Wu et al., 2012; Iguchi et al., 2013; Su et al., 2014)	*ALS2, ANG, CHMP2B, DCTN1, EWSR1, ERBB4, FIG4, hnRNPA1, MATR3* (Matrin-3), *OPTN, PFN1, SETX, SIGMAR1, SQSTM1, SPG11, TAF15, UBQ2N2, VAPB, VCP*	Protein degradation; ER-Golgi pathways; Trafficking; Endosomal sorting complexes required for transport; DNA and RNA processing; Actin dynamics; Mitogenesis and differentiation

*Proteins are indicated in parenthesis for some cases: SOD1, Superoxide Dismutase 1; TDP-43, TAR DNA-binding protein 43; FUS, Fused in Sarcoma protein*.

## Calcium Dysregulation in ALS-Affected Motor Neurons—The Factual Case of ALS1

A particular feature that distinguishes ALS affected MN from other cells relates to their inherent vulnerability to Ca^2+^ overload; indeed, these neurons highly express Ca^2+^ permeable AMPA receptors (Williams et al., 1997; Shaw and Eggett, 2000; Van Den Bosch et al., 2000; Vandenberghe et al., 2000; Guatteo et al., 2007) concurrently with a low Ca^2+^ buffering capacity due to endogenous low expression of Ca^2+^ buffering proteins (CaBPs) such as parvalbumin and calbindin (Alexianu et al., 1994; Palecek et al., 1999; Jaiswal, 2013) albeit the presence of EF-hand Ca^2+^ binding proteins in MN (Migheli et al., 1999; Zhang et al., 2014). This combination of inherent physiological features of MN to manage Ca^2+^ levels, though essential for normal functioning (von Lewinski and Keller, 2005), are likely a predisposition risk for the systematic intracellular Ca^2+^ overload that is detected in ALS1 affected MN (Siklós et al., 1996, 1998; Kruman et al., 1999; Grosskreutz et al., 2010; Kawamata and Manfredi, 2010). Interestingly, the levels of calretinin and parvalbumin in MN axons is found further decreased in ALS patients (Hayashi et al., 2013), thus establishing an increased deficit in MN Ca^2+^ buffering capability under pathological conditions.

In fact, a direct outcome of the low expression of CaBPs in MN is that mitochondria are likely to assume a major role in buffering calcium in these cells. In agreement, it might not be a coincidence that in ALS1, mutated SOD1 was shown to abnormally accumulate in the mitochondrial intermembrane space (Jaarsma et al., 2001; Liu et al., 2004), affecting mitochondrial function leading to disturbance of Ca^2+^ homeostasis and ERMCC cycle (Jaiswal and Keller, 2009; Lautenschläger et al., 2013). Moreover, studies on cellular and animal ALS1 models have shown that Ca^2+^ overload is linked with SOD1 aggregation (Tateno et al., 2004; Tradewell et al., 2011). Indeed, we have recently shown that Ca^2+^ can bind to SOD1 immature states promoting its aggregation (Leal et al., 2013; Estácio et al., 2015) thus establishing an additional ALS1 pathological pathway for the impact of Ca^2+^ overload on SOD1 toxic deposition. In agreement, a decrease of intracellular Ca^2+^ overload in ALS1 models through AMPA channel antagonists or overexpression of calcium-buffering proteins has been shown to reduce SOD1 toxic aggregation and neurodegeneration (Beers et al., 2001; Van Damme et al., 2003; Tateno et al., 2004; Tortarolo et al., 2006; Yin et al., 2007; Parone et al., 2013).

## Crossways of ALS Critical Proteins with Calcium

In spite of the scarcity of available data regarding more recently discovered fALS models linked with systematic Ca^2+^ deregulation, compelling evidence argues that many critical proteins involved in ALS (other than SOD1), are directly or indirectly involved with Ca^2+^. It is noteworthy that in such instances, ALS-associated mutations in many of these ALS critical proteins happen to potentiate Ca^2+^ deregulation and/or result in an increased vulnerability to the effects of Ca^2+^. For example, VAP-B which is involved in ALS8, is a ER-membrane MAM protein directly engaged in Ca^2+^ exchange between the ER and mitochondria (De Vos et al., 2012). The ALS VAPBP56S variant was shown to disrupt Ca^2+^ homeostasis leading to a perturbation of the anterograde mitochondrial axonal transport and affecting the Miro1/kinesin-1 interaction with tubulin (Mórotz et al., 2012). Alsin, a protein implicated in juvenile ALS2, is involved in endossome/membrane trafficking that undergoes Ca^2+^ dependent binding to the NCS regulating neurocalcin alpha protein (Masutani et al., 2008). This suggests that alsin membrane binding might be a Ca^2+^ dependent process, and therefore passible to become affected by dysregulation of Ca^2+^ levels. Moreover, alsin is also found to play a role in AMPAR trafficking, where ALS2 mutations lead to distinct subcellular GRIP1 localization and reduction of the calcium-impermeable GluR2 containing AMPA receptors, thus likely rendering neurons susceptible to deviant Ca^2+^ influxes (Lai et al., 2006). Matrin 3, a multifunctional nuclear matrix protein involved in ALS21, is suggested to be regulated through a Ca^2+^ dependent interaction with CaM (Valencia et al., 2007) and is therefore likely to be affected by deregulated Ca^2+^ levels. As only very recently matrin 3 has been implicated in ALS (Johnson et al., 2014), future studies will be needed to clarify this possibility. Ataxin-2, which is involved in ALS13 is an ubiquitous cytoplasmic protein proposed to induce defects in the ER–Golgi pathway and disrupt Ca^2+^ signalling (van den Heuvel et al., 2014). The possibility that ataxin-2 influences ER–Golgi function is inferred from a recent study where it was shown that intermediate-length polyQ expansions in ataxin-2 mutants enhance FUS-induced ER stress and Golgi fragmentation (Farg et al., 2013). The suggestion that the ALS13 related intermediate-length polyQ expansions in ataxin-2 can lead to Ca^2+^ signalling disruption derives from the causal association that polyQ-expanded forms of ataxin-2, or huntingtin and ataxin-3 in other diseases, associates with the C-terminal domain of the intracellular calcium release channel receptor—InsP_3_R1 and enhance InsP_3_R1-mediated calcium release in neurons (Tang et al., 2003; Chen et al., 2008; Liu et al., 2009). Thus, it is tempting to speculate that intermediate-length polyQ expansions in ataxin-2 may also cause it to associate with InsP_3_R1 and thereby modulate calcium signaling, though this was not yet shown. In ALS6, the FUS protein leads to CAMK2N2 up-regulation (Convertini et al., 2013), an inhibitor of CAMKII that regulates neuronal synaptic plasticity through phosphorylation of AMPA receptors. Given that it has been shown that abnormal CAMKII inhibition by small molecules and peptides results in dysregulation of Ca^2+^/glutamate signalling (Ashpole et al., 2012), it may be hypothesized that CAMKII inhibitor—CAMK2N2 up-regulation by mutant FUS will also result in Ca^2+^ dysregulation in ALS6. Moreover, deregulated Ca^2+^ levels can activate the Ca^2+^-dependent calpain protease that cleaves TDP-43 at the C-terminal, generating aggregation prone N-terminal segments that are found misallocated in the majority of ALS patients (even in those that do not carry TDP-43 mutations associated with ALS10) driving TDP-43 toxicity across ALS pathology (Aggad et al., 2014; Yamashita and Kwak, 2014). In addition, TDP-43 is simultaneously found to interact with other critical proteins in ALS namely matrin-3 (Johnson et al., 2014), ataxin-2 (Nihei et al., 2012), VAPB (Stoica et al., 2014), and SOD1 (Volkening et al., 2009) and is therefore rather tempting to conjecture about a tight interrelation between Ca^2+^ dyshomeostasis and the involvement of critical proteins in ALS.

## ALS Toxic Processes and the Role of Calcium

In fact, major pathological processes in ALS involving excitotoxicity and the ER-mitochondria Ca^2+^ cycle are deeply connected and potentially trigger or/and are enhanced by intracellular Ca^2+^ deregulation: (a) Glutamatergic excitotoxicity is tough to be mediated by an excessive influx of extracellular ions, including Ca^2+^, resulting in elevated intracellular Ca^2+^ levels that can activate cytoplasmatic Ca^2+^-dependent apoptotic proteins (e.g., calcineurin, calpain) which promote cell death (Wang et al., 1999; Kim et al., 2002); (b) elevated intracellular levels of Ca^2+^ also lead to mitochondrial Ca^2+^ overload, that is deeply interconnected with mitochondrial dysfunction resulting in ROS production, oxidative stress and eventually to apoptosis or necrosis (Kawamata and Manfredi, 2010; Cozzolino and Carrì, 2012); and (c) depletion of Ca^2+^ levels in the ER which is suggested to occur via a persistent shift of Ca^2+^ from the ER to the mitochondria due to deregulated ER MCC leads to protein folding dysfunction and proteasome impairment, resulting in ER stress and apoptosis (Prell et al., 2013; Tadic et al., 2014). Mutations in critical proteins associated with ALS actually seem to increase the susceptibility for these toxic processes to occur. For example, misfolded and aggregated SOD1 mutants localized within the mitochondrial membrane of spinal cord MN cause dysfunction in oxidative phosphorylation and bind aberrantly to Bcl-2, generating toxicity (Jung et al., 2002; Mattiazzi et al., 2002; Liu et al., 2004; Vande Velde et al., 2008; Pedrini et al., 2010); also, the ALS linked P56S mutation in VAPB, or th A4V, G85R and G93A SOD1 mutations leads to toxic protein aggregation and ER stress (Prosser et al., 2008; Kim et al., 2010; Atkin et al., 2014).

## Conclusions

Overall, we here argue that Ca^2+^ deregulation seems to establish a converging point for major ALS dysfunctional pathways and critical associated proteins, and can therefore be a key environmental factor to better understand ALS etiology and its pathomechanisms. However, we do not intend to ground that all proteins implicated in ALS will necessarily lead to Ca^2+^ deregulation; rather, we seek to discuss that processes involving Ca^2+^ could directly or indirectly (e.g., via Ca^2+^ effects on processes dependent of other divalent cations) account for their mutual involvement in ALS. Interestingly, the so-called “calcium hypothesis” establishing a close link between Ca^2+^ deregulation and neurodegeneration, has also been suggested to play a central role in other neurodegenerative disorders such as Alzheimer’s, Ataxia, Parkinson’s and Huntington Diseases (Bezprozvanny, 2010; Kasumu and Bezprozvanny, 2012), where Ca^2+^ channels and proteins involved in neuronal Ca^2+^ signalling systems are likely potential targets for therapeutic strategies (Zundorf and Reiser, 2011).

## Conflict of Interest Statement

The authors declare that the research was conducted in the absence of any commercial or financial relationships that could be construed as a potential conflict of interest.
